# 非小细胞肺癌新辅助治疗联合外科治疗的进展

**DOI:** 10.3779/j.issn.1009-3419.2017.05.09

**Published:** 2017-05-20

**Authors:** 亚旗 王, 兴 王, 石 阎, 跃 杨, 楠 吴

**Affiliations:** 100142 北京，北京大学肿瘤医院暨北京市肿瘤防治研究所胸外二科，恶性肿瘤发病机制及转化研究教育部重点实验室 Key Laboratory of Carcinogenesis and Translational Research (Ministry of Education), Department of Thoracic Surgery Ⅱ, Peking University Cancer Hospital & Institute, Beijing 100142, China

**Keywords:** 肺肿瘤, 新辅助治疗, 手术, Lung neoplasms, Neoadjuvant therapy, Surgery

## Abstract

肺癌是世界范围内发病率和死亡率最高的恶性肿瘤。对于可手术切除的Ⅲa/N2期非小细胞肺癌患者，目前国内外指南均推荐采用手术联合化疗、放疗等多学科治疗模式。最新研究表明，与术后辅助治疗一样，新辅助治疗（化疗或放化疗）可显著改善可切除非小细胞肺癌患者的预后，且在治疗依从性及耐受性方面具有明显优势。非小细胞肺癌新辅助治疗的对象主要是局部进展期病变，特别是临床Ⅲa/N2期患者，基本治疗模式为术前2-4周期化疗，新辅助治疗后并不增加手术相关的死亡及并发症风险，但是在决定手术时机、入路及切除范围等方面仍面临着挑战。

## Abstract (en)

Background and objective Lung cancer is the leading form of cancer in terms of both incidence and cancer-related deaths. For patients with resectable Ⅲa/N2 non-small cell lung cancer (NSCLC), guidelines in and abroad recommend multidisciplinary team treatment, including surgery and chemotherapy, radiotherapy or other comprehensive treatment. Newly published evidences prove that neoadjuvant therapy can improve outcomes of NSCLC patients significantly, with advangtages in tolerability and compliance medication. Neoadjuvant therapy has been adopted mainly in locally advanced NSCLC, especially in stages Ⅲa/N2 patients, and chemotherapy of 2-4 cycles has become the basic pattern. Neoadjuvant therapy does not increase the concomitant complications of chemotherapy and surgery. However, challenges still exist in determining subsequent surgical timing, approach and extent of resection.

肺癌是世界范围内发病率和死亡率最高的恶性肿瘤，其中非小细胞肺癌（non-small cell lung cancer, NSCLC）占80%-85%^[1, 2]^。据统计，约有1/3的NSCLC确诊时已处于局部进展期（Ⅲ期）^[3]^。对于可手术切除的Ⅲa/N2期NSCLC，虽然近年来手术技术的进步使围术期并发症大大降低，但单纯手术切除术后5年生存率仍为20%-35%^[4]^，且术后复发率及远处转移率高，因此目前国内外指南均推荐采用手术联合化疗、放疗等多学科治疗模式，基本治疗策略已经从手术切除+术后辅助含铂双药方案化疗^[5, 6]^逐渐过渡到根治性同步放化疗或者新辅助治疗后再行根治性手术等综合治疗方案。近20年来，新辅助治疗在NSCLC综合治疗中的地位一直存在争论。最新证据表明，与术后辅助治疗类似，新辅助治疗也可显著改善可切除NSCLC患者的预后，且安全、可行，并不增加化疗及手术相关的并发症。现就NSCLC新辅助治疗联合外科治疗的一些研究进展综述如下。

## 1 新辅助治疗的理论基础及适应人群

### 1.1 新辅助治疗的理论基础

1982年，Frei等^[7]^首先提出了新辅助化疗的概念，主要应用于头颈部癌、骨肿瘤、乳腺癌等实体肿瘤，指恶性肿瘤局部治疗（手术或放疗）前给予的全身或局部化疗，也称术前化疗（preoperative chemotherapy），用以区别于术后辅助化疗。理论上，新辅助治疗存在以下优势：①使肿瘤缩小、分期降低；②提高手术的可切除性；③消灭或预防术前可能存在的微转移；④较之术后患者提高了化疗的耐受性；⑤术前因肿瘤血供保持完整，化疗药物更有效地到达病灶；⑥可测病灶的存在提供了活体药敏检测的效果^[8]^。

### 1.2 新辅助治疗的适应症

最新2016年版NSCLC NCCN指南推荐，新辅助治疗的主要适应人群是Ⅲa期患者如T3侵犯胸壁、T4侵犯纵隔结构或者气管、肺上沟瘤（T3-4N0-1）以及T1-3/N2病变。有些临床试验也纳入较早期肺癌包括Ⅰb期、Ⅱ期以及有纵隔淋巴结微小转移的“偶然性N2”的Ⅲa期肺癌患者^[9-11]^。

## 2 新辅助治疗的研究进展

### 2.1 新辅助化疗联合手术对比单纯手术

1994年，Roth和Rosell分别发表了两项经典的Ⅲa期NSCLC新辅助化疗的前瞻性随机对照研究。Roth等^[12]^将60例Ⅲa期NSCLC患者随机分为术前3周期的CEP方案（环磷酰胺、依托泊苷、顺铂）新辅助化疗组和单纯手术组。两组不完全切除或不可切除的患者接受术后放疗。结果发现新辅助化疗组的中位生存时间明显延长（64个月*vs* 11个月，*P* < 0.008）。Rosell等^[13]^得到相似的结论，研究同样入组60例Ⅲa期NSCLC患者，随机分为术前3周期的MIP方案（异环磷酰胺、丝裂霉素C、顺铂）新辅助化疗组和单纯手术组，术后均接受放疗，新辅助组中位生存时间为22个月，3年、5年生存率分别为20%和17%，单纯手术组中位生存时间为10个月，3年、5年生存率分别是5%和0%。这两项研究均显示术前化疗组比单纯手术组有明显的生存获益，且具有统计学意义，从而奠定了新辅助化疗在Ⅲa期NSCLC多学科综合治疗中的地位。

法国DePirre等^[9]^随后报道了一项纳入早期NSCLC的多中心前瞻性随机对照研究（MIP-91），共有355例Ⅰ期（T1N0除外）、Ⅱ期及可手术的Ⅲa期患者入组。患者被随机分为术前2周期MIP方案新辅助化疗组和单纯手术组，化疗有反应者再给予2周期术后辅助化疗，如果病理结果为T3、N2或不完全切除者同时给予术后放疗。新辅助化疗组的病理完全缓解率为11%，部分缓解率为53%。结果显示，术前化疗组中位生存时间有延长趋势（37个月*vs* 26个月），但是没有统计学差异（*P*=0.15）。在中位无病生存期（disease-free survival, DFS）方面，术前化疗有明显优势（26.7个月*vs* 12.9个月，*P*=0.033），且远处转移率也低于单独手术组（*P*=0.01）。该研究结果并没有达到预期收益，究其原因，可能与化疗方案的选择有关。值得注意的是，研究亚组分析显示对于无纵隔淋巴结转移的Ⅰ期-Ⅱ期（N0-1）患者，术前化疗的生存获益更明显（*P*=0.027），而对Ⅲa期（N2）患者并没有明显优势（*P*=0.85）；对于化疗后达到缓解特别是病理完全缓解的患者，死亡风险明显降低（RR=0.42, *P*=0.000, 1），然而遗憾的是目前并没有相关研究来识别这一类患者。

基于前述的研究结论，Scagliotti等^[11]^设计一项前瞻性的随机对照研究——CHEST（早期肺癌化疗试验）。研究入组270例Ⅰ期（T1N0除外）、Ⅱ期及部分Ⅲa期（T3N1：除外肺上沟瘤）NSCLC患者，随机分为术前3周期新辅助化疗组和单纯手术组。化疗药物为顺铂和吉西他滨。结果显示，新辅助化疗组的无进展生存期（progression-free survival, PFS）（HR=0.70, *P*=0.03）和总生存期（overall survival, OS）（HR=0.63, *P*=0.02）明显延长。但是亚组分析显示，对于Ⅰb/Ⅱa期患者，两组的PFS（HR=1.06, *P*=0.83）和OS（HR=1.02, *P*=0.94）均无明显差异。新辅助化疗的生存优势主要体现于Ⅱb/Ⅲa期患者（3年PFS率：55.4% *vs* 36.1%，*P*=0.002；OS：HR=0.42，*P* < 0.001）。该研究结果提示新辅助化疗更适于分期较晚（Ⅱb或Ⅲa期）的可切除NSCLC，而对于偏早期（Ⅰb期或Ⅱa期）患者，目前的临床观点仍不能统一。

然而，也有一些临床研究结果质疑新辅助化疗的疗效。日本临床肿瘤组（Japan Clinical Oncology Group, JCOG）开展了一项比较术前化疗和单纯手术的前瞻性随机对照研究。研究纳入1993年至1998年共62例Ⅲa/N2期NSCLC患者，随机分为新辅助化疗组和单纯手术组。结果显示，新辅助化疗对Ⅲa/N2期NSCLC患者在生存时间上没有获益^[14]^。但是这项研究因入组率低而过早终止，从而大大降低了结果的统计功效。Gilligan等^[10]^开展的一项前瞻性随机对照试验得到同样的结果。该研究入组了来自70个中心的519例NSCLC患者，其中大部分（61%）为Ⅰ期，31%为Ⅱ期，7%为Ⅲ期。所有患者被随机分配到新辅助化疗组和单纯手术组。结果显示，新辅助化疗并不能增加NSCLC患者的总体生存（中位生存时间：54个月vs 55个月，*P*=0.86）。

鉴于早期的临床研究结论不一，且缺乏多中心、大样本的临床随机对照研究（表 1）。2006年，Burdett等^[15]^对包括988例NSCLC患者的7个随机对照试验进行meta分析，发现新辅助化疗在可切除NSCLC患者中有生存获益，5年生存率增加了6%，但是受样本量及试验数目的限制，结论的说服力显得有些不足。

**1:** 新辅助化疗对比单纯手术组的临床研究 Trials of preoperative chemotherapy followed by surgery versus surgery in non-small cell lung cancer

Author [year]	Number of patients	Clinical stage	Preoperative chemotherapy	Response rate	Postoperative chemotherapy cycles planned	Postoperative radiotherapy planned	Median OS（wks/ys）	Median D FS or PFS（wks/ys）
Roth *et al* [1994]	60	Ⅲa	Cyclophosphamide, etoposide, cisplatin; 3 cycles every 4 week	35%	3 to responders	Yes (if surgery incomplete or unresectable)	PCT: 64 wks S: 11 wks *P* < 0.008	*
Rosell *et al* [1994]	60	Ⅲa	Mitomycin, ifosfamide, cisplatin; 3 cycles every 3 weeks	60%	0	Yes	PCT: 26 wks S: 8 wks *P* < 0.001	PCT: 20 wks S: 5 wks *P* < 0.001
Depierre *et al* [2002]	355	Ⅰb-Ⅲa	Mitomycin, Ifosfamide, cisplatin; 2 cycles every 3 weeks	64%	2 to responders	Yes (if surgery incomplete or pT3 or pN2)	PCT: 37 wks S: 26 wks *P*=0.15	PCT: 26.7 yrs. S: 12.9 yrs. *P*=0.033
Nagai *et al* [2003]	62	Ⅲa	Vindesine, cisplatin; 3 cycles every 4 weeks	28%	0	Yes (if surgery Incomplete)	PCT: 17 wks S: 16 wks *P*=0.527	*
Gilligan *et al* [2007]	519	Ⅰ-Ⅲ	One of six platinum-based regimens#; 3 cycles every 3 weeks	49%	0	Yes (if surgery incomplete or progression)	PCT: 54 wks S: 55 wks *P*=0.86	PCT: 26 wks S: 25 wks *P*=0.74
Scagliotti *et al* [2012]	270	Ⅰb-Ⅲa	Gemcitabine, cisplatin; 3 cycles every 3weeks	35.4%	0	No	PCT: 7.8 yrs. S: 4.8 yrs. *P*=0.04	PCT: 4.0 yrs S: 2.9 yrs *P*=0.09
OS: overall survival; DFS: disease free survival; PFS: progression free survival; PCT: preoperative chemotherapy; S: surgery; wks: weeks; yrs, years; one of six platinum-based regimens#: Mitomycin, vinblastine, cisplatin; or mitomycin, ifosfamide, cisplatin; or vinorelbine, cisplatin; or paclitaxel, carboplatin; or gemcitabine, cisplatin; or docetaxel, carboplatin; *: not provided.								

2014年，NSCLC荟萃分析协作组发表在*Lancet*杂志上的一篇以单个患者数据（individual participant data, IPD）为基础的*meta*分析文章，共纳入了包括2, 385例NSCLC患者的15项随机对照试验，结果显示NSCLC新辅助化疗对比单纯手术生存获益显著，相对的死亡风险降低13%（HR=0.87, 95%CI : 0.78-0.96, *P*=0.007），绝对5年生存获益大约在5%（从40%提高到45%）。Ⅰb期-Ⅲa期患者无复发生存率（HR=0.85, *P*=0.002）以及远处转移的时间（HR=0.69, *P* < 0.000, 1）均显著提高，同时局部复发的时间也有延长趋势（HR=0.88, *P*=0.20），但没有统计差异^[16]^。这项研究肯定了新辅助化疗在可手术切除NSCLC患者综合治疗中的重要地位。

另外一项研究对包括3, 324例NSCLC患者的13项随机对照试验进行*meta*分析，同样发现NSCLC新辅助化疗组的OS优于单纯手术组（HR=0.84, 95%CI: 0.77-0.92, *P*=0.000, 1）^[17]^。

### 2.2 新辅助化疗对比术后辅助化疗

术后辅助治疗已经被证实可以延长患者生存，但是术后患者合并症和功能恢复差的问题常常阻碍辅助化疗的实施。NATCH Ⅲ期临床试验研究较早比较新辅助化疗和辅助化疗在Ⅰ期-Ⅲa期NSCLC综合治疗中的地位，该项前瞻性研究共入组624例Ⅰ期、Ⅱ期和部分Ⅲa期(T3N1)患者，随机分为单独手术组（212例）、新辅助化疗+手术组（201例）以及手术+辅助化疗组（211例）。结果显示，与辅助化疗相比，新辅助化疗有更好的依从性，97%新辅助化疗患者完成治疗计划，而仅66.2%术后辅助化疗患者完成治疗计划（*P* < 0.000, 1）；三组的5年生存率分别为34.1%、38.3%和36.6%，新辅助化疗组的生存获益优于其他两组，但是没有统计学差异（*P*=0.71）^[18]^。

NATCH试验并没有明确新辅助化疗与辅助化疗在NSCLC患者治疗中的优势。该研究进一步讨论导致阴性结果的原因，一方面可能是入组对象以Ⅰ期患者为主（75.1%为cⅠ期，49.8%为pⅠ期），另一方面，化疗药物是紫杉醇和卡铂，并没有采用有效性更好的顺铂。值得注意的是，研究的亚组分析显示，Ⅱ期-Ⅲa期患者接受新辅助化疗后5年生存率明显改善（新辅助化疗组36.6%对比术后辅助化疗组31%、单纯手术组25%）。

2016年10月，在丹麦首都哥本哈根召开的欧洲临床肿瘤协会年会（European Society of Clinical Oncology Annual Meeting, ESMO）上，吴一龙教授^[19]^汇报了CSLC0501研究的最终结果。该项多中心、前瞻性Ⅲ期临床试验同样比较新辅助化疗和术后辅助化疗对NSCLC患者的生存影响，共纳入来自13个治疗中心的214例Ⅰb期-Ⅲa期可切除的NSCLC患者，主要终点指标为3年DFS率，次要终点指标为3年、5年OS率以及安全性。最终共有198例患者符合入组条件，其中Ⅰb期、Ⅱa期、Ⅱb期和Ⅲa期患者的比例分别为32.5%、12.2%、28.4%和26.9%，随机分为两组：新辅助化疗组（97例）、辅助化疗组（101例）。化疗方案为DC方案（多西他塞+卡铂）。结果显示，新辅助化疗组的全部患者（100%）完成化疗，而辅助化疗组仅87.4%完成化疗。两组患者在治疗过程均未出现预料外的毒性反应，41.2%的患者发生3级-4级中性粒细胞减少，辅助化疗组有1例化疗相关的死亡，新辅助化疗组有1例死于围术期肺栓塞。新辅助化疗组和辅助化疗组的3年DFS率分别为43.0%和56.0%（HR=0.76, *P*=0.172），两组差异无统计学意义。两组3年OS率分别为64.0%和68.0%（HR=0.88, *P*=0.602），5年OS分别为43.0%和60.0%（HR=0.66, *P*= 0.049），均无显著统计学差异。

虽然CSLC0501研究亦是阴性结果，新辅助化疗并未优于辅助化疗。但是研究至少证实，对于Ⅰ期-Ⅲa期NSCLC，两种治疗策略都是可行且安全的。鉴于新辅助化疗在治疗可及性方面具有明显优势，对于局部晚期估计手术根除困难或者预计术后不能耐受辅助化疗等特殊人群，可以考虑行新辅助化疗，以期得到最大的生存获益。

### 2.3 术前化疗对比围术期化疗

目前已有的数据显示无论术前化疗还是术后化疗，在NSCLC综合治疗中均可带来显著的生存获益。那么，化疗应采用何种方式与手术相结合是一个值得探索的问题。IFCT0002研究对比了术前2周期+术后2周期和术前2-4周期方案，共入组528例Ia期-Ⅱ期NSCLC患者，随机分为新辅助化组和围术期化疗组。结果发现，新辅助化疗组（术前2-4周期）和围术期化疗组（术前2周期+术后2周期）并没有显著生存差别（3年OS率：67.8% *vs* 68.6%，*P*=0.96），而且新辅助2周期和新辅助4周期在病理缓解率方面也无统计学差异^[20]^。

Burdett等^[16]^纳入的15项随机对照试验中，10项试验仅行术前化疗，5项试验在手术前后均行化疗，术前化疗周期为2-3周期。结果显示，无论化疗在术前进行还是手术前后都进行，目前的证据没有提示这两种治疗模式对生存获益有影响（交互作用*P*=0.23），而且术前化疗周期数对生存获益也无影响（交互作用*P*=0.68）。

基于上述研究，新辅助化疗和围术期化疗在生存获益方面没有显著差别，这也提示新辅助化疗并没有因手术延迟而影响治疗效果。

### 2.4 术前单用化疗对比术前联合放化疗

2015年，Pless等^[21]^为研究新辅助化疗中加入放疗能否改善NSCLC患者生存获益而开展一项前瞻性的Ⅲ期随机研究，共纳入了2001年至2012年23个中心的232例T1-3N2的Ⅲa/N2期NSCLC患者，随机分为新辅助化疗组和新辅助放化疗组。全组中位随访时间52.4个月。结果显示，新辅助化疗和新辅助放化疗的两组患者的中位无病生存期（11.6个月*vs* 12.8个月）及总生存期（26.2个月*vs* 37.1个月）均无明显统计学差异。对比新辅助放化疗与新辅助化疗，Ⅲa/N2期NSCLC患者加入放疗并没有显著改善新辅助化疗联合手术的生存效益，而且两组的病理完全缓解率和淋巴结降期率相似。

国内学者针对新辅助放化疗的生存获益进行一项*meta*分析，共纳入了包括2, 724例Ⅲ期NSCLC患者的12项临床试验，其中8项为随机对照试验，4项为回顾性研究。结果同样显示新辅助放化疗在生存获益方面并不优于新辅助化疗。但是对比单纯新辅助化疗，新辅助放化疗对于肿瘤降期（*P*=0.01）、纵隔淋巴结的完全缓解率（*P*=0.028）、局部控制率（*P*=0.002）有意义^[22]^。

目前证据显示，新辅助放化疗对比新辅助化疗并不能显著改善NSCLC患者的生存获益，而且其在病理缓解率和淋巴结降期率方面的作用，有必要期待新的随机对照试验进一步分析。

### 2.5 综合治疗模式的争议：放疗、化疗联合手术三联模式对比放疗联合化疗两联模式

对于部分Ⅲa/N2期NSCLC患者，传统的治疗手段为根治性放化疗，但局部复发率较高。前述的研究已经证实新辅助治疗联合手术能够改善可切除Ⅲa/N2期NSCLC患者的的生存获益。Albain等^[23]^进行的一项前瞻性Ⅲ期临床试验比较新辅助放化疗联合手术与标准的放化疗（即三联模式对比两联模式）对Ⅲa/N2患者的生存影响。研究共纳入429例NSCLC患者，均接受了EP方案的同步放化疗，后随机分为三联治疗组和两联治疗组，最终共396例符合入组条件，其中202例为三联治疗组，194例为两联治疗组。三联治疗组共有155例患者最终接受手术，3例为楔形切除术，98例为肺叶切除术，54例为全肺叶切除术。结果显示，三联治疗组的无进展生存期（progression free survival, PFS）优于两联治疗（12.8个月*vs* 10.5个月，*P*=0.017），两者的总生存期（overall survival, OS）没有显著区别（23.6个月*vs* 22.2个月，*P*=0.24）。有趣的是，在亚组分析中，与两联治疗相比，肺叶切除组显示出一定的生存获益（OS）（33.6个月*vs* 21.7个月，*P*=0.002）。

2015年，McElnay等^[24]^发表一项的*meta*分析共纳入6项随机对照研究，868例确诊N2的NSCLC患者进行诱导化疗或者诱导放化疗，之后随机进行手术或者非手术治疗。结果显示，进行手术的三联治疗模式对比非手术的两联治疗模式有生存获益（HR=0.87, 95%CI: 0.75-1.01, *P*=0.068），虽然没有传统意义上的统计学差异，但是通过对点估计值和可信区间的分析可推测，前者的总生存率相对获益13%。

临床共识认为，N2期NSCLC诱导治疗后降期可考虑后续手术，但是若N2仍阳性，则认为手术没有生存获益。然而最近Massard等^[25]^发表的一篇综述性文章认为，对于新辅助治疗后持续N2的NSCLC，若能完全切除，手术仍不失为一种很好的治疗选择。文章通过对几篇关于N2期NSCLC研究的介绍得到这一结论。Port等^[26]^在2005年最早研究手术对于新辅助治疗后N2仍阳性的NSCLC的意义，52例手术患者中仅19例（36%）经新辅助治疗后降期，所有手术患者5年生存率为23%，N2降期者为30%，N2仍阳性者为19%，多因素分析显示，N2阳性并不是重要的预后因素（*P*=0.65）。2009年，来自Leuven的一项报道^[27]^，共85例患者新辅助治疗后接受手术切除，认为新辅助治疗后N2降期与否对生存获益无明显影响。Mansour等^[28]^发表的一项研究入组153例行全肺切除的患者，其中28例（18.3%）新辅助治疗后N2阳性，32例（20.9%）N2降期，93例（60.8%）未接受新辅助治疗，结果显示，三组的5年生存率分别为32.2%，34.8%和12.4%，研究认为全肺切除手术可适用于新辅助治疗后N2仍阳性的NSCLC。

因此对于可切除Ⅲa/N2期NSCLC，手术应当成为其综合治疗不可或缺的部分。值得注意的是，现实世界中，欧洲与北美地区对于此类患者的处理策略上又有些不同。北美学者偏爱术前诱导治疗，常常将手术限定于诱导治疗降期的N2患者，且全肺切除的比例偏低；而欧洲学者则更支持手术在综合治疗中的重要地位，更倾向于将手术放在各种治疗手段之前，此外N2患者手术比例是北美地区的两倍，且全肺切除比例也较高^[29]^。2010年美国国立综合癌症网络（National Comprehensive Cancer Network, NCCN）成员单位进行了一项关于N2患者调查问卷，90.5%认为对于单站纵隔淋巴结转移，小于3 cm，可考虑手术，而对于多站纵隔淋巴结转移（3 cm以下），仅47.6%认为可考虑手术。

### 2.6 T3侵犯胸壁、T4侵犯纵隔结构或者气管、肺上沟瘤（T3-4N0-1）的新辅助治疗

这些特殊类型的肺癌并不常见，导致目前缺乏其相关的大型前瞻性研究或荟萃分析。对于可手术切除且无纵隔淋巴结转移的肺上沟瘤（T3-4N0-1），NCCN指南推荐采用新辅助放化疗联合根治性手术的治疗模式。Rusch等^[30]^开展的一项前瞻性多中心研究入组111例肺上沟瘤（N0-1）患者，其中80例（72.1%）为T3病变，31例为T4病变。所有患者术前接受2周期的同步放化疗。治疗有反应或者病变稳定者3到5周后接受手术治疗（肺叶切除联合受侵胸壁的整块切除）。结果显示，102例（92%）患者完成同步放化疗，其中95例最终接受手术，76例（92%）为完全性切除，54例（65%）表现为术后病理完全缓解或残留微小病变；所有接受手术的患者的2年生存率为55%，其中完全性切除者更是高达70%。研究的长期随访结果显示，所有手术患者的5年生存率为44%，完全性切除者为54%，而且肿瘤复发主要发生在远隔部位^[31]^。因此，术前同步放化疗可提高肺上沟瘤的手术可切除率，改善患者的长期生存。

而对于肿瘤侵犯胸壁、气管或纵隔的T3-4患者（N0-1），临床判断可完全性手术切除的病变，目前NCCN指南倾向于首选手术联合辅助化疗的治疗模式，然而也有学者认为术前也可选择新辅助化疗或者新辅助同步放化疗^[32]^。临床上判断不可切除的部分T4N0-1病变是否可采用新辅助治疗联合手术的治疗策略存在较多争议，尚无明确指南推荐。然而最新ESPATUE研究显示，部分不可切除的T4N0-1病变经过诱导化疗或放化疗后，T分期明显降期，转变为可手术切除，且有明显的长期生存获益^[33]^。因此，对于这类患者在初始治疗时应进行有效的多学科会诊，以制定个体化的治疗策略。

## 3 新辅助治疗后手术面临的挑战

美国国家癌症数据库（National Cancer Data Base, NCDB）最新分析显示，在多学科综合治疗的Ⅲa期NSCLC患者中，大约15%接受了新辅助放化疗和手术^[34]^。

### 3.1 新辅助治疗后手术风险问题

Brouchet等^[35]^研究新辅助化疗对NSCLC患者术后并发症的影响，回顾性地分析了2002年6月-2004年6月来自法国51个胸外科中心的3, 888例肺癌手术患者的临床资料，其中555例（14.3%）接受新辅助化疗。研究采用*Logistic*回归分析患者术前的临床特征与主要的术后并发症的关系，结果显示术后住院死亡率为3.01%，多因素分析年龄（ > 65岁）、性别（男性）、术前临床评分（中高危）、手术方式（右全肺切除和联合肺叶切除）是围术期死亡的风险因素，但是对比新辅助化疗与否，两组间无统计学差异；共1, 219例患者（31.4%）有至少一项术后并发症，最常见的是肺不张（7.1%）、漏气（6.8%）、肺炎（4.2%）、心律失常（3.4%）、出血（2.2%）、急性呼吸窘迫综合征（1.8%）、喉返神经麻痹（1.7%）、瘘（1.1%），多因素分析显示新辅助化疗对术后并发症率无显著影响。

Glover等^[36]^发表一项回顾性研究，52例临床证实N1或N2且新辅助治疗后的NSCLC患者行机器人辅助肺叶切除术（robotic-assisted video thoracoscopiclobectomy, RAVTL），其中39例未接受术前治疗，7例接受新辅助化疗，以及6例接受新辅助放化疗。结果发现，与单纯手术相比，新辅助化疗或者新辅助放化疗并不增加病人的术中出血量、手术时间以及住院死亡率，并且各组围术期总并发症率没有明显差异。研究证实新辅助治疗对比单纯肺叶切除手术，并没有增加患者的术中风险及术后并发症。

Pless等^[21]^发现，新辅助放化疗与单纯新辅助化疗在患者术后并发症方面也没有明显区别，甚至是对于行全肺切除的患者（44例，占全部病例的23%）。另一项研究，Veronesi等^[37]^入组55例接受支气管成型术的肺癌患者，其中27例（49%）接受新辅助治疗（24例为化疗，3例为放化疗），28例（51%）未接受术前治疗。结果显示，两组患者术后均无死亡，新辅助治疗组有2例出现主要并发症（7.4%），6例出现轻微并发症（22%），而单纯手术组有1例出现主要并发症（3.6%），9例出现轻微并发症（32%），两组的主要及轻微并发症率均无统计学差异。因此新辅助治疗（化疗或放疗）后进行支气管成形术也是安全可行的。

### 3.2 新辅助治疗后的手术时机

目前关于NSCLC新辅助治疗后手术时机的选择的研究很少。Gao等^[38]^最近发表一项回顾性研究，尝试探索新辅助放化疗后至手术的不同时间间隔对总生存率的影响。研究收集了来自美国国家癌症数据库2004年-2012年间共1, 623例Ⅲa期（T1-3N2）NSCLC患者的资料。依据诱导治疗后至手术的时间间隔将患者分成4组（即0周-3周，3-6周，6-9周以及9-12周），各组所占比例分别是7.9%、50.5%、31.9%、9.6%。手术方式为全肺切除、肺叶切除或者亚肺叶切除术。多因素生存分析显示时间间隔在6周之内时总生存率没有显著差异（*P*=0.107），但是当超过6周时，即6周-9周（*P*=0.043）和9周-12周（*P*=0.030）两组的总生存率均显著下降。而且进一步分析发现，接受全肺切除的患者的生存获益明显差于肺叶切除，尽管6周-9周和9周-12周两组的患者接受全肺切除的比例明显低于0周-3周的患者（14.3% *vs* 22.5%, *P*=0.026; 12.1% *vs* 22.5%, *P*=0.021），但最终生存并没有显示出优势，提示时间间隔可能对各组生存率的影响更大。

研究结果为NSCLC新辅助治疗后选择手术的时机提供了一定的参考。但是，该研究为回顾性分析，不同手术间隔的分组亦为非随机性，这可能导致潜在的选择偏倚，而且研究并没有提及各组患者的手术方式存在显著差异的原因。这些因素都有可能影响最终结果的判断。因此，我们期待更多的前瞻性非随机对照研究来证实这一结果。

### 3.3 手术入路的问题：开放或者微创

临床Ⅲa期病变采用微创手术治疗缺乏前瞻性的随机对照研究。传统意义上认为的一些微创手术的相对禁忌症，例如较大肿瘤，中心型肿瘤，侵犯邻近结构或者淋巴结转移，新辅助化放疗后，已经在一些中心通过技术改进得以实现。

一项回顾性研究提出与微创手术相比，开放手术清扫的淋巴结数目更多。该研究分析了2008年-2012年间共129例行肺叶切除的cN0肺癌患者，其中69例（53.5%）接受开放手术，60例（46.5%）接受微创手术。结果显示前者清扫的淋巴结数目显著多于微创手术[(14.7±1.3) *vs* (9.9±0.8), *P*=0.003）]；开放手术组中，24.6%的患者术后病理升期为pN1或pN2，而微创组仅为10%（*P*=0.05）^[39]^。然而，Zhong等^[40]^认为两者在清扫的淋巴结数目上没有差别，研究共纳入157例行肺叶切除的cN0期NSCLC患者，且术后病理证实为pN2，其中67例接受微创手术，90例接受放手术。结果显示，两种手术方式清扫的总淋巴结数目相似[(17.4±6.1) *vs*(18.1±7.2), *P*=0.78]，且纵隔淋巴结清扫数目也无差异[(11.7±5.6) *vs* (12.0±5.1), *P*=0.84]。

目前有多项非随机对照研究回顾性地比较微创与开放手术对Ⅲa期患者长期生存率的影响。Yang等^[41]^共入组621例NSCLC患者，其中67例为Ⅲa期或更晚分期。13%患者最终接受微创手术，其余为开放手术。结果显示，Ⅲa期行微创手术的5年生存率为22%，而开放手术为24%。因此，手术入路并不影响Ⅲa期NSCLC生存获益。但该研究样本较少，且属于非随机对照研究，证据可靠性欠佳。

在2015年的美国临床肿瘤学会（American Society of Clinical Oncology, ASCO）会议上，Woodard介绍他的中心倾向于在Ⅲa状态下行开放手术。他们认为开放手术有利于彻底的淋巴结清扫，并提供最好的局部控制率。但微创手术也有其适应症与价值^[42]^。

### 3.4 手术的切除范围：全肺切除或者袖式肺叶切除

#### 3.4.1 新辅助化疗后全肺切除的现状

新辅助治疗是否增加全肺切除手术风险的问题在业内存在很大争议。最早Fowler等^[43]^报道了一项回顾性研究，13例局部进展期NSCLC患者经新辅助放化疗后接受肺叶切除或者全肺切除，结果显示术后有6例肺叶切除无一例出现术后死亡，但7例全肺切除术后有3例死亡（43%），且全肺切除术后的并发症率也高达62%。研究认为，新辅助治疗明显增加全肺切除术的术后并发症率和死亡率，但因样本量受限，结论欠缺说服力。日本学者Matsubara等^[44]^也得到相似结论，新辅助治疗后行术后并发症率和死亡率分别为63.2%和7.0%。

但有些学者得到相反的结论。2001年，Siegenthaler等^[45]^发现新辅助化疗后的8例全肺切除无一例死亡，而28例单纯全肺切除者有3例死亡，且前者术后并发症率明显低于单纯手术（*P*=0.026）。另一项回顾性研究^[46]^，共纳入298例行全肺切除的NSCLC患者，其中60例为新辅助化疗组（20.1%），238例为单纯手术组（79.9%）。结果显示，新辅助化疗组和单纯手术组的术后30 d死亡率分别为6.7%和5.5%（*P*=0.458），术后90 d死亡率分别为11.7%和10.9%（*P*=0.512）；两组术后并发症率如脓胸（1.7% *vs* 2.1%, *P*=0.458），支气管胸膜瘘（1.7% *vs* 5.5%, *P*=0.188），急性呼吸窘迫综合征（3.3% *vs* 3.4%, *P*=0.675）均无统计学差异。相比于肺叶切除术而言，新辅助治疗可能没有增加全肺切除术的死亡风险和合并症发生的风险。

2012年，一项系统综述和荟萃分析纳入27个研究^[47]^，分析比较新辅助治疗后全肺切除术30 d及90 d死亡率和合并症的发生情况。结果显示，新辅助治疗后行全肺切除术总体30 d及90 d死亡率分别为7%和12%，左全肺切除和右全肺切除的30 d死亡率分别为5%和11%，最主要的死因是肺部并发症如支气管胸膜瘘等。荟萃分析显示，与左全肺切除相比，右全肺切除的30 d死亡率显著升高（OR=1.97; 95%CI: 1.11-3.49, *P*=0.02），同样右全肺切除的90 d死亡率也显著升高（OR=2.01; 95%CI: 1.09-3.72, *P*=0.03）；该研究显示，新辅助治疗后右全肺切除的死亡风险明显高于左全肺切除，而且全肺切除术后30 d及90 d的死亡率差异（5%）明显超出预期，提示术后30 d死亡率并不能完全反映出围术期的死亡情况。因此在新辅助化疗后如仍需行右全肺切除术应格外慎重。

#### 3.4.2 肺切除术的替代方式——袖式切除术

对于部分中心型肺癌，为最大限度地保留残余肺功能，新辅助治疗后可采用袖式肺叶切除，从而避免行全肺切除。

有研究认为新辅助化疗并没有增加袖式肺叶切除术的并发症率及死亡率^[48]^。Gonzalez等^[49]^同样认为诱导化疗或诱导放化疗后袖式切除的围术期死亡率和术后并发症率都与非诱导治疗组相似，并且前者并没有带来较多的吻合口并发症。Storelli等^[50]^更是认为，新辅助治疗后的袖式切除即使不进行吻合口包裹处理也是安全的。

关于长期疗效，前述研究并没有发现新辅助化疗联合袖式手术和直接袖式手术在长期生存方面的差异，两组的中位生存期为43.1个月和33.5个月，差异无统计学意义。该研究还比较新辅助治疗后袖式切除与全肺切除的生存差异，结果显示，新辅助治疗后袖式切除术5年生存率优于全肺切除（73.4% *vs* 33.3%, *P*=0.000, 65）^[48]^。

由此看来，对于部分中心型肺癌，只要外科技术上能够实现袖式吻合，新辅助治疗后应首选袖式肺叶切除。与全肺切除术相比，它并不增加术后并发症率和死亡率，且有较好的长期生存获益和生活质量。

### 3.5 老年患者诱导治疗后的手术问题

年龄被认为是非小细胞肺癌术后并发症率和死亡率的一个重要危险因子^[51, 52]^。随着世界人口老龄化的加速，临床实践中会遇到越来越多的高龄肺癌患者。对待此类患者，特别是可耐受诱导治疗的高龄肺癌患者，外科医师需要面临后续手术带来的相关问题。2016年，一项回顾性研究分析了高龄（≥70岁）对新辅助治疗后NSCLC患者的围术期并发症和长期生存的影响。研究共入组317例患者，年龄大于或等于70岁的为53例（中位年龄为74岁），45%进行诱导化放疗，最终均接受肺叶或全肺切除术。结果显示，高龄组（≥70岁）与低龄组（ < 70岁）术后30天死亡率相似，分别为6%和5%，两者术后的并发症率亦无明显区别（57% *vs* 49%, *P*=0.30），并且两者中位总生存率也无统计学差异^[53]^。

Marquez-Medina等^[54]^也得到相似的结论，研究共入组108例患者，高龄组（≥70岁）与低龄组（ < 70岁）分别为44例和64例，其中50例患者经新辅助治疗后接受手术（全肺切除、肺叶或亚肺叶切除）。结果显示，低龄组与高龄组总生存率、无病生存率方面无明显差异。

因此，对于经过详细术前筛查评估的高龄患者，新辅助治疗后行肺叶或全肺切除术是安全可行的。

## 4 小结

近年的临床研究肯定了新辅助化疗或新辅助放、化疗在可切除NSCLC综合治疗中的重要地位。NSCLC新辅助治疗的对象主要是局部进展期病变，特别是临床Ⅲa/N2期患者；对临床Ⅰ期和Ⅱ期患者的治疗作用，新辅助治疗尚需积累更多的数据供临床决策。
